# Predictive Role of Systemic Inflammatory Indices in Surgically Managed Postpericardiotomy Syndrome Following Cardiac Surgery

**DOI:** 10.3390/diagnostics15121488

**Published:** 2025-06-11

**Authors:** Murat Yücel, Emrah Uğuz, Muhammet Fethi Sağlam, Kemal Eşref Erdoğan, Mete Hıdıroğlu, Altay Alili, Şeref Alp Küçüker

**Affiliations:** 1 Department of Cardiovascular Surgery, Ankara Bilkent City Hospital, Ankara 06800, Türkiye; alilialtay97@gmail.com (A.A.); serefalp@yahoo.com (Ş.A.K.); 2 Department of Cardiovascular Surgery, Faculty of Medicine, Ankara Yıldırım Beyazıt University, Ankara 06010, Türkiye; emrahuguz@gmail.com (E.U.); dr.m.fethisaglam@gmail.com (M.F.S.); kemal_esref@hotmail.com (K.E.E.); metetaha@hotmail.com (M.H.)

**Keywords:** pericarditis, postoperative complications, biomarkers, postpericardiotomy syndrome, cardiac surgery

## Abstract

**Objective:** This study aimed to evaluate the prognostic utility of systemic inflammatory markers, such as the Systemic Immune-Inflammation Index (SII), Systemic Inflammatory Response Index (SIRI), Neutrophil–Lymphocyte Ratio (NLR), Monocyte–Lymphocyte Ratio (MLR), and Platelet–Lymphocyte Ratio (PLR), to identify patients at risk of developing surgically treated postpericardiotomy syndrome (PPS). **Methods:** A total of 150 patients were retrospectively analyzed. In total, 75 patients who developed postpericardiotomy syndrome requiring surgical drainage constituted the postpericardiotomy group, whereas 75 age- and surgically matched non-PPS patients served as the control group. Blood samples were collected at four time points: preoperative (T1), 24 h postoperative (T2), 7 days postoperative (T3), and 24 h before secondary intervention in the PPS group and the closest matched outpatient follow-up (T4) in the control group. Inflammatory marker values were compared within and between the groups at the four defined time points. Logistic regression and receiver operating characteristic (ROC) analyses were used to determine the diagnostic and predictive accuracy of each marker. **Results:** Significant increases in the SIRI, MLR, and CRP levels were observed in patients who developed PPS and required surgical intervention. MLR on postoperative day 7 had the highest sensitivity (84%) with a cut-off of 0.575, whereas SIRI demonstrated the highest specificity (81.3%) at a cut-off of 3.34. SII increased significantly only in the late stage, indicating disease progression. The NLR lacked predictive power across all time points. **Conclusions:** The SIRI and MLR are promising early-stage biomarkers for identifying patients at high risk of developing PPS. Their integration into routine postoperative follow-up could facilitate earlier diagnosis and reduce surgical burden. A multi-marker approach may enhance the diagnostic precision of PPS beyond that of traditional inflammatory measures.

## 1. Introduction

Postpericardiotomy syndrome (PPS), a subtype of post-cardiac injury syndrome, is characterized by autoimmune pericarditis that typically develops weeks to months after myocardial infarction, cardiac surgery, or cardiac trauma [1]. The reported incidence of PPS varies widely between the past and present. Previous studies have reported an incidence of 10–30% in adult cardiac surgery patients [2,3]. In recent studies, the incidence has been reported to range from 21% to 9% [4]. Although the clinical picture is well-characterized, the underlying pathophysiological mechanisms remain unclear. It is thought to result from an autoimmune response triggered by myocardial or pericardial injury [5]. These autoantibodies accelerate the inflammatory cascade, resulting in pericardial and pleural inflammations.

The clinical presentation of PPS ranges from mild self-limiting pericarditis to severe pericardial effusion, which requires invasive intervention [6]. Clinically, pericarditis presents as pleuritis, fever, and chest pain [5]. Typically, the median time to the resolution of PPS after cardiac surgery is 2–3 weeks [7,8]. Possible recurrences tend to occur within 2–11 weeks of the initial onset [9].

Despite significant advances in cardiac surgery and perioperative care, it remains a clinical challenge, and its incidence varies widely depending on surgical technique and patient characteristics [10]. While current guidelines emphasize NSAIPPS, colchicine, and corticosteroids for treatment [11], a proportion of patients remain at risk for persistent inflammation and recurrent effusions requiring surgical drainage [12].

Currently, no specific biomarker or standardized diagnostic criteria can reliably predict the development or severity of PPS at an early stage. In addition, the lack of an objective distinction between mild and severe forms of PPS makes the early prediction of high-risk cases difficult. Predictive factors for PPS, especially differentiating between patients who require invasive intervention and those who do not, have not been adequately investigated. Although traditional inflammatory markers such as the C-reactive protein (CRP) and erythrocyte sedimentation rate (ESR) are usually elevated in PPS, they lack specificity and predictive accuracy in the postoperative period [13].

The Systemic Inflammatory Response Index (SIRI) and Systemic Inflammation Index (SII) are novel biomarkers that reflect the interplay between the immune and inflammatory processes. They have been used to determine the degree of inflammation in many clinical conditions, especially in oncology, cardiovascular diseases, and autoimmune diseases [12]. However, their application in the context of PPS has yet to be explored. Given that PPS is a putative inflammatory condition with an autoimmune component, these markers may provide information regarding the onset, progression, and severity of the disease. This study provides a novel analysis to understand the dynamics and potential of systemic inflammatory response by integrating novel biomarkers such as SIRI and SII into the clinical evaluation of PPS. By evaluating the dynamic changes in these biomarkers from the preoperative to the postoperative phase, we aimed to identify reliable markers that can facilitate early diagnosis and intervention and to identify factors leading to the need for repeat surgical drainage. Thus, we hope to contribute to the development of predictive models for early diagnosis and individualized therapeutic approaches.

## 2. Materials and Methods

### 2.1. Study Design and Patient Selection

This retrospective observational cohort study was conducted at our center between January 2019 and January 2025. The study population consisted of patients aged 18–80 years who underwent cardiopulmonary bypass (CPB)-assisted cardiac surgery. As the majority of patients requiring surgical intervention for PPS underwent on-pump cardiac surgery, only those who underwent CPB were included to ensure homogeneity and to rule out the heterogeneous effects of on-pump and off-pump techniques on systemic inflammation. Informed consent was not obtained owing to the retrospective nature of the study.

A total of 150 patients were included in this study. The study population was divided into two groups. The first group (*n* = 75), referred to as the control group, included patients who experienced an uncomplicated postoperative course without inflammatory complications or the need for further surgical intervention following cardiac surgery. The second group (*n* = 75), termed the PPS group, comprised patients who underwent a second invasive intervention, such as subxiphoid drainage, thoracotomy, median sternotomy, or pericardiocentesis, due to a diagnosis of PPS occurring on or after the 14th postoperative day. Although all patients were diagnosed after day 14, the wide standard deviation in the timing of surgical drainage (29.05 ± 17.48 days) reflects interindividual variability in the interval between PPS diagnosis and the decision for surgical intervention, without inclusion prior to day 14. When forming the control group, stratification was applied based on the type of surgical intervention performed to reduce possible selection bias. The control group patients were divided into subgroups according to the surgical procedures performed, and patients with a surgical profile similar to that of the PPS group but who were not diagnosed with PPS were identified. Then, one-to-one (1:1) patient file matching was performed using a random selection method by non-clinical personnel (floor secretary) without any clinician intervention or guidance among these files. Randomization was performed without knowledge of inflammation, the development of complications, or prognostic information in the patient file. This approach aimed at minimizing the surgical heterogeneity between the case and control groups and reducing the risk of selection bias.

**Figure 1:**
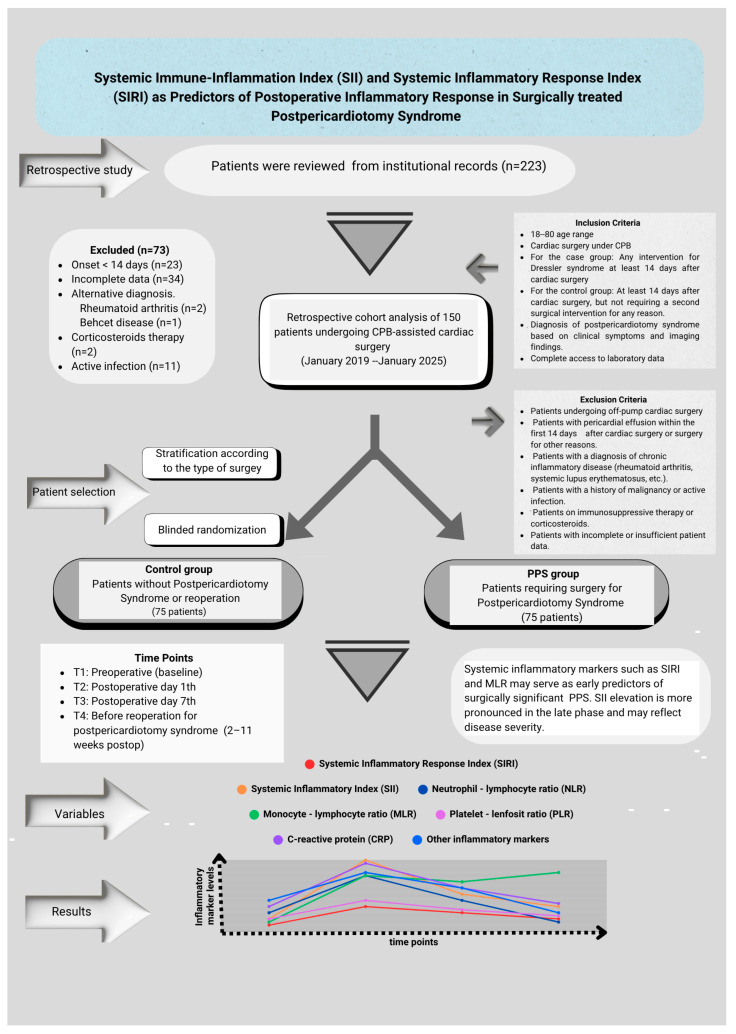
Flow diagram showing the study design, patient selection, and matching process. From a total of 223 cardiac surgery patients screened, 73 were excluded based on predefined criteria (early onset of symptoms, incomplete data, or alternative diagnosis). The remaining 150 patients were divided into two groups (*n* = 75 each), PPS and control groups, following stratification and blinded random selection. Abbreviations: CPB, cardiopulmonary bypass; PPS, postpericardiotomy syndrome; SIRI, Systemic Inflammatory Response Index; SII, Systemic Immune-Inflammation Index.

The inclusion and exclusion criteria for the study design are shown in Figure 1. Patient identification was performed by reviewing medical records, operative notes, and hospital information system (HIS) data. The study was approved by the Ethics Committee of Ankara Bilkent City Hospital, No. 1 (Approval No: 652, 23 October 2024). All the procedures were conducted in accordance with the principles of the Declaration of Helsinki. Confidentiality of personal data was meticulously protected, and patient identities were anonymized.

### 2.2. Diagnosis of Postpericardiotomy Syndrome

In accordance with the available literature and ESC (European Society of Cardiology) guidelines, only patients who developed PPS–14–90 days after cardiac surgery were included in this study [7,8,9,14]. Pericardial effusion and inflammatory responses that develop in the first two weeks following surgery are generally considered to be associated with acute postoperative inflammation or surgical complications (e.g., bleeding, tamponade, and infection), and these patients were excluded.

The diagnosis of PPS is based on a combination of clinical symptoms, electrocardiographic changes, laboratory findings, and imaging findings. The clinical symptoms include fever, pericardial pain, and pericardial friction. Elevated levels of inflammatory markers, such as the C-reactive protein (CRP) and erythrocyte sedimentation rate (ESR), along with electrocardiographic findings indicative of pericarditis, are key components of the diagnosis. Telecardiography was performed routinely. However, in cases of clinical suspicion, transthoracic echocardiography (TTE) and/or chest computed tomography (CT) are performed. Significant pericardial effusion was defined as pericardial fluid depth surrounding the heart at >10 mm, particularly in the right and left ventricular regions and signs of cardiac compression. Indications for invasive intervention in patients are determined using a standard clinical protocol and assessment. After the diagnosis of PPS, all patients were started on standard medical treatment in accordance with the current ESC guidelines. This treatment includes a combination of first-line anti-inflammatory agents such as nonsteroidal anti-inflammatory drugs (NSAIPPS) (e.g., ibuprofen 600–800 mg/day or indomethacin 50 mg/day (every 12 h) and colchicine (0.5–1.0 mg/day). Corticosteroids were not used as the initial treatment. In cases where, despite optimal medical treatment and progressive or recurrent pericardial effusion, the echocardiographic findings of tamponade compromised hemodynamic stability or refractory clinical symptoms were observed, invasive intervention was decided. Cardiac tamponade was diagnosed on the basis of clinical findings (heart rate > 100/min or paradoxical pulse [a decrease in systolic blood pressure of >10 mmHg during inspiration]) and echocardiographic evidence of right ventricular compression during diastole echocardiography [15]. However, shortness of breath, persistent fever, and persistently elevated inflammatory markers such as CRP and WBC were the main determinants of the decision for surgical drainage. The duration of preoperative medical treatment varied according to the clinical response and imaging findings; however, all surgical cases represented medically resistant or worsening PPS.

### 2.3. Data Collection and Measurements

Peripheral blood samples were collected at four different time points to evaluate the temporal changes in inflammatory markers in both groups. These points were defined as T1, the preoperative period; T2, the first 24 h postoperatively; T3: the 7th postoperative day; and T4, 24 h before the intervention for PPS in the PPS group and the closest matched outpatient clinic follow-up day in the control group.

The SII, SIRI, Neutrophil–Lymphocyte Ratio (NLR), Monocyte–Lymphocyte Ratio (MLR), Platelet-to-Lymphocyte Ratio (PLR), C-reactive protein (CRP) level, and leukocyte (WBC) count were analyzed in both groups. The inflammatory indices were calculated using the following formulae:

- Systemic Immune-Inflammation Index (SII) = (Platelet count × Neutrophil count)/Lymphocyte count.
- Systemic Inflammatory Response Index (SIRI) = (Neutrophil count × Monocyte count)/Lymphocyte count.

Comparisons were made both within and between the groups at four time points. Blood samples were collected according to standard and time-controlled protocols before surgery, within the first 24 h after surgery, and on the 7th day after surgery. However, in the group of patients who underwent surgery due to PPS, biomarkers for the fourth time point (preoperative) were collected at heterogeneous time intervals between the 2nd and 11th weeks postoperatively, when the disease required clinical surgery. This limited the temporal matching of data from the fourth time point between the PPS and control groups.

To improve temporal comparability, T4 measurements in the control group were selected from routine follow-up visits scheduled according to our institutional protocol at 10 days, 4 weeks, and 8 weeks after surgery. This approach aimed to obtain values aligned with the average time distribution in the PPS group to ensure clinical and temporal equivalence. Although this method does not completely eliminate the risk of temporal mismatch, it allows for a meaningful clinical comparison of systemic inflammation trends at comparable postoperative stages. Therefore, methodological consistency was achieved in both cohorts using standardized follow-up intervals.

The inflammatory markers from T3 were used to predict PPS. This time point was chosen because the acute postoperative inflammatory response was largely due to systemic surgical trauma, whereas the inflammatory profiles emerging around day 7 were more specific and likely to reflect pathological processes.

### 2.4. Operative Management and Drainage Procedure

All the patients diagnosed with pericardial tamponade were transferred to the operating room. The choice of incision for pericardial fluid drainage was determined by the surgical team on the basis of echocardiography (Philips EPIQ Philips Healthcare, Andover, MA, USA) and/or CT findings. All patients underwent general anesthesia. Perioperative antibiotic prophylaxis and drainage management at our institution were conducted according to standardized institutional protocols, which are in alignment with the international perioperative care guidelines published by the ESC and the European Association for Cardio-Thoracic Surgery (EACTS). The decision to open a pericardiopleural window (PPW) was made by the same surgical team.

At the end of the procedure, a closed tube drainage system was placed in the pericardial or thoracic region to drain pericardial fluid. The drainage tube remained in the pericardial or thoracic cavity for at least 72 h, and the drainage volume was closely monitored. The drain was removed when the daily drainage volume fell below 100 cc or when follow-up echocardiography showed that the remaining fluid volume was less than 5 mm in both the anterior and posterior regions. Repeat echocardiography or CT evaluation was performed for suspected tamponade or hemodynamic instability.

### 2.5. Statistical Analysis

In the power analysis performed using G*Power Version 3. 1. 9. 7 software, a medium effect size (Cohen’s f = 0.25), alpha = 0.05, and power = 0.80 for at least 64 patients were required in each group. However, 75 patients were included in each group to obtain reliable results. Continuous variables were expressed as the mean ± standard deviation and categorical data were reported as frequencies and percentages. An independent sample *t*-test was used to compare the differences between the two groups for normally distributed data, and the Mann–Whitney U test was used for non-normally distributed data. The chi-square test was used to compare categorical data. To examine longitudinal changes over time, a repeated-measures analysis of variance (ANOVA) was used for normally distributed variables, while the Friedman test was used for non-normally distributed variables. The Wilcoxon Signed-Rank Test was applied in the post hoc analysis to determine the differences over time.

Univariate logistic regression analysis was used to evaluate the association between inflammatory markers and PPS development and to analyze the risk factors a priori. Statistically significant variables (*p* < 0.05) were included in the multivariate regression analysis to determine independent risk factors. For multivariate analyses, a stepwise model was created using the Forward Likelihood Ratio (LR) method. Odds ratios (ORs) with 95% confidence intervals (CIs) were calculated using a logistic regression analysis. The overall fit and validity of the model were assessed using the Hosmer–Lemeshow fit test. A receiver operating characteristic (ROC) curve analysis was performed to evaluate the effectiveness of inflammatory markers in predicting the development of PPS. The results are presented as the area under the curve (AUC). Inflammatory markers that predicted PPS occurrence and corresponded to the maximum value of the Youden index were used to determine optimal cut-off values. A *p* value < 0.05 was considered significant in all statistical analyses

The Statistical Package for the Social Sciences, version 27.0, was used for the statistical analysis of the data obtained. Microsoft^®^ Excel^®^ MSO for Microsoft 365 (Version 2503 Build 16.0.18623.20116) was used alongside 64 bits for the tables, Microsoft Visio Professional 2019 for graphics and images, and the Canva Pro program (www.canva.com, (accessed on 1 April 2025)) for flow charts.

## 3. Results

The demographic and preoperative characteristics of the study population are shown in Table 1. The mean age was significantly lower in the PPS group than in the control group (54.88 ± 14.33 vs. 60.00 ± 10.89 years, *p* = 0.025). There was no statistically significant difference in sex distribution between the groups (*p* = 0.473), with males constituting 73.33% of the control group and 68% of the PPS group. The mean body weight and body mass index (BMI) were also significantly lower in the PPS group compared to the control group (75.41 ± 11.54 vs. 80.88 ± 11.92 kg, *p* = 0.010; and 26.43 ± 4.57 vs. 28.26 ± 4.39 kg/m^2^, *p* = 0.004, respectively). The preoperative left ventricular ejection fraction (LVEF) did not significantly differ between the two groups (55.79 ± 7.91 vs. 54.95 ± 8.77, *p* = 0.666). The chronic disease and comorbidity profiles of patients in both groups were similar. An analysis of the blood group distribution showed that Rh-positive A was the most prevalent blood group in both groups, followed by Rh-positive A. However, no significant differences in the ABO and Rh distributions were detected between the groups (*p* = 0.977).

The mean time from primary cardiac surgery to the second surgical intervention for PPS was 29.05 ± 17.48 days (median: 21 days). The operative characteristics of the study cohort are summarized in Table 2. Cardiopulmonary bypass (CPB) and aortic cross-clamp (ACC) times were comparable between the two groups, with no statistically significant differences.

However, the distribution of primary surgical procedures differed significantly. Aortic valve replacement (AVR) was the most frequently performed procedure in the control group (*n* = 14, 18.7%), whereas aortic surgery was the most prevalent in the PPS group (*n* = 29, 38.7%) (*p* = 0.003). Sternotomy was the most common surgical approach in both groups, with no significant difference in frequency (*p* = 0.620).

Regarding postoperative drainage, mediastinal chest tube placement alone was the most common approach in both groups (control: *n* = 35, 46.7%; PPS: *n* = 30, 40%). Multicompartmental drainage, defined as the insertion of tubes into the mediastinum, left thorax, and right thorax, was more frequently used in the PPS group (*n* = 11, 14.7%) than in the control group (*n* = 6, 8.0%); however, the difference was not statistically significant (*p* = 0.518).

The early postoperative outcomes in both groups are summarized in Table 3. There were no statistically significant differences between the PPS and control groups in terms of the length of intensive care unit (ICU) stay, the duration of intubation, or total postoperative drainage volume. Similarly, the number of transfused blood products and the incidence of early postoperative complications did not differ significantly between the two groups (*p* > 0.05).

However, patients in the PPS group had a significantly longer total hospital stay than those in the control group (*p* < 0.05). Additionally, the rehospitalization rate was significantly higher in the PPS group, suggesting a greater burden of postoperative morbidity. Although the 30-day mortality rate was higher in the PPS group (*n* = 6, 8%), the difference was not significant (*p* = 0.058).

A comparison of postoperative anticoagulant and antiplatelet strategies, as well as the mean international normalized ratio (INR) values between the study groups, is presented in Table 4. The INR levels reflect the spot measurements obtained preoperatively and before corrective intervention in the PPS group. In the control group, the INR values were retrieved during routine outpatient follow-up visits. Among the patients receiving warfarin therapy, the mean INR was higher in the PPS group (2.87 ± 0.93) than in the control group (2.52 ± 0.48); however, this difference was not statistically significant (*p* = 0.114).

There were no statistically significant differences between the two groups in terms of anticoagulant or antiplatelet use. Warfarin was the most commonly used agent in both groups (59.5% in the control group vs. 47.7% in the PPS group, *p* = 0.32). The use of acetyl salicylic acid (ASA), clopidogrel, dual antiplatelet therapy (DAPT; ASA + clopidogrel), triple therapy (TT; DAPT + anticoagulant), and direct oral anticoagulants (DOACs) was also comparable between the groups (*p* > 0.05).

The surgical drainage methods used in patients requiring invasive intervention for PPS are summarized in Table 5. Four different surgical approaches were used in this cohort, with statistically significant variations in their distribution (*p* < 0.001). Thoracotomy (right or left) was the most frequently performed technique (37 patients, 49.3%), followed by median sternotomy (26 patients, 34.7%). Pericardiocentesis under echocardiographic guidance was applied in seven patients (9.3%), while subxiphoid incision was used in only five patients (6.7%).

In addition, 33 of 75 patients (44.0%) underwent a pericardiopleural window (PPW) procedure in addition to thoracotomy or sternotomy according to intraoperative findings and effusion characteristics.

Telecardiography was routinely used to image all patients with PPS (*n* = 75). In the diagnostic and invasive intervention decision-making process, the combined use of CT and echocardiography was preferred over other methods (*n* = 35, 46.67%), followed by echocardiography alone (*n* = 24, 32%) and CT alone (*n* = 16, 21.33%). When the agreement between the effusion amounts obtained from CT and echocardiography imaging methods was analyzed with the intraclass correlation coefficient (ICC) coefficient, no agreement was found between the two methods (ICC = 0.155, CI:95% (−0.170; 0.449 and *p* = 0.173).

Table 6 shows the temporal changes in systemic inflammatory markers (SII, SIRI, NLR, PLR, MLR, CRP, and WBC) at four predefined time points (T1–T4) in both the control and PPS groups. Significant between-group differences over time were observed for all markers except NLR (*p* < 0.001 for all other markers). A significant increase in the SII, SIRI, PLR, CRP, and WBC counts was noted in the PPS group, especially at T4, as the time point before surgery. These levels were significantly higher in the PPS group than in the control group at the same time point (*p* < 0.05).

**Figure 2:**
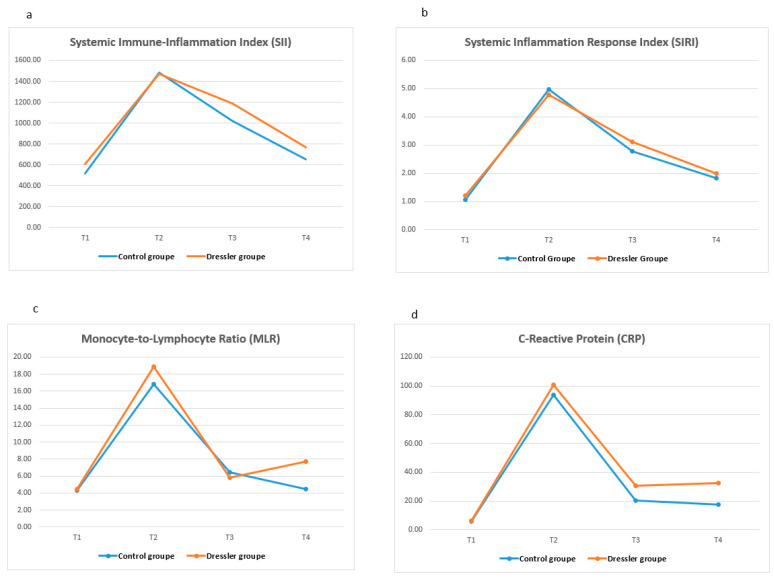
Temporal changes in systemic inflammatory markers across four predefined time points: T1 (preoperative), T2 (24 h postoperative), T3 (7 days postoperative), and T4 (24 h before surgical intervention in the PPS group or matched outpatient visit in the control group). A comparison between the PPS and control groups is shown for (**a**) the Systemic Immune-Inflammation Index (SII); (**b**) the Systemic Inflammatory Response Index (SIRI); (**c**) Monocyte-to-Lymphocyte Ratio (MLR); and (**d**) C-reactive protein (CRP). Statistical significance (between-group *p* values) are as follows: T3: SIRI (*p* = 0.036), MLR (*p* = 0.001), and CRP (*p* = 0.093); T4: SIRI (*p* = 0.064), MLR (*p* = 0.106), and CRP (*p* < 0.001).

In the PPS group, the SII, PLR, and CRP levels were significantly elevated at T4 but not at earlier time points. In contrast, the SIRI and WBC counts started to increase at T3 and peaked at T4, indicating a progressive systemic inflammatory response. Furthermore, while MLR was significantly higher in the PPS group at T3, this difference was no longer significant at T4. There was no significant difference in NLR at any time point in either group. Dynamic changes in inflammatory markers at four different time points are shown as a linear graph in Figure 2.

Table 7 summarizes the temporal changes in standard biochemical parameters, including urea, creatinine, lactate dehydrogenase (LDH), aspartate aminotransferase (AST), alanine aminotransferase (ALT), and albumin, measured at the four time points. LDH levels were significantly higher at T4 in the PPS group (*p* = 0.020), whereas no significant differences were found between the groups for the other markers. The within-group differences over time were not statistically significant. 

Univariate and multivariate logistic regression analyses were performed to determine the independent variables that were effective for predicting the development of PPS cases that required invasive intervention. Table 8 presents the results of the analysis. Univariate logistic regression analysis revealed a significant differential effect of age, BMI, monocyte count, MLR, and CRP level in blood collected on postoperative day 7 (*p* < 0.05). Parameters estimated to be significant in the univariate analysis were included in the multivariate analysis. In the multivariate analysis, age, monocyte count at T3, and the CRP level were statistically significant predictors in the reduced model using the Forward Likelihood Ratio (LR) method (*p* < 0.05). Age alone was found to have a statistically significant effect on predicting PPS in both univariate and multivariate analyses, independent of other variables.

**Figure 3:**
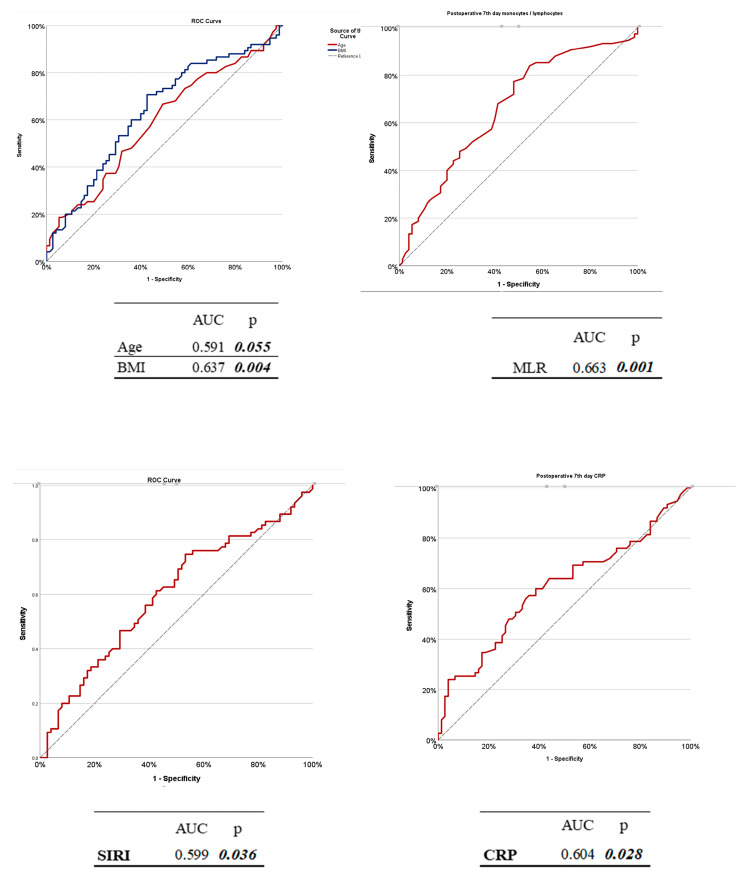
Receiver operating characteristic (ROC) curves for biomarkers selected to predict surgically significant postpericardiotomy syndrome measured on day 7 post-surgery (T3). The curves represent the age, SIRI, CRP level, and MLR. The AUC and *p* values for these variables are provided in detail in Table 9.

To evaluate the diagnostic performance of the selected variables for predicting the development of PPS (PPS) cases requiring invasive intervention, receiver operating characteristic (ROC) analyses were performed for age, SIRI, CRP, and MLR using data collected at T3 (postoperative day 7). The results are summarized in Table 9, and the corresponding ROC curves are shown in Figure 3. Among the evaluated variables, MLR demonstrated the highest discriminatory power with an AUC of 0.663 (*p* = 0.001). A cut-off value of 0.310 yielded a sensitivity of 84.00% and a specificity of 45.33%. The CRP level also showed a statistically significant predictive value (AUC = 0.604, *p* = 0.028), with a sensitivity of 60.00% and specificity of 61.33% at a threshold of 16.47 mg/L. The SIRI provided a modest but statistically significant discriminatory ability (AUC = 0.599, *p* = 0.036) with a cut-off value of 3.34, yielding a sensitivity of 32.00% and a relatively high specificity of 81.33%. In contrast, age had a poor predictive performance (AUC = 0.460, *p* = 0.055), indicating limited utility in isolation. The best cut-off value for age was 57 years, with 48.00% sensitivity and 64.00% specificity.

## 4. Discussion

PPS has long been recognized as a delayed-onset autoimmune or inflammatory complication of cardiac surgery. However, considering the non-specificity of inflammatory parameters in the diagnostic process in these studies and the physiologic variability of the postoperative period, it can be said that the current criteria are insufficient to adequately differentiate true clinical cases of PPS [16]. Currently, both the diagnostic criteria and clinical management remain controversial. To date, there have been no comprehensive studies in the literature regarding the progression of PPS and the dynamic changes in inflammatory indices. Despite advances in perioperative care and surgical techniques, the pathophysiological mechanisms underlying PPS progression, especially those requiring invasive drainage, have not been adequately elucidated [12]. This study aimed to analyze temporal changes in systemic inflammatory indices in PPS cases requiring invasive intervention, perform early risk classification, and develop predictive models for the development of PPS cases requiring invasive procedures. Thus, high-risk patients can be identified earlier, and immunomodulatory treatments can be initiated sooner in patients with elevated inflammatory markers. This may potentially reduce the need for surgery in PPS. Our study demonstrated that among the inflammatory indices analyzed, MLR on postoperative day 7 was the most sensitive predictor of surgically treated PPS, whereas SIRI showed the highest specificity. CRP levels were also significantly correlated with PPS development, particularly in the later stages. These findings suggest that dynamic changes in inflammatory markers may offer predictive value in identifying high-risk patients early in the postoperative course.

Multiple risk factors have been associated with the development of PPS, including younger age [17] and female sex [18], which are frequently cited in the literature. Females are known to be more prone to autoimmune inflammatory diseases. This is thought to be related to hormonal, genetic, and environmental factors [19]. However, some large-scale epidemiological studies have reported conflicting results regarding the effect of sex, which may be due to differences in patient selection and surgical procedures [18]. In our study, no statistically significant association was found between sex and PPS occurrence (*p* = 0.473), supporting the view that sex alone may not be a reliable predictor in this population.

In contrast, age appeared to be a more consistently observed risk factor. In a large prospective study, the incidence of PPS was 18% among adults aged ≥ 21 years but declined to 10% in individuals aged > 70 years [20]. Similar trends were reported by Miller et al., with the PPS incidence decreasing progressively from 24% in patients aged < 54 years to 11% in those aged over 65 [17]. It has also been reported that autoimmune responses are more active in young individuals; therefore, immune hyperactivity may lead to exaggerated inflammatory responses [21,22]. Although the general incidence of autoimmune diseases increases with age [23], some autoimmune processes are activated in younger age groups [21]. Therefore, younger ages may be more susceptible to the development of PPS. In contrast, age-related changes in the immune system, especially a decrease in T and B lymphocyte activity, may cause an inadequate immune response [24]. In our study, the fact that the mean age was significantly lower in the patients who developed PPS is consistent with these findings. However, in the ROC analyses, the discriminative power of the age variable was weak (0.055), the most appropriate cut-off value was determined to be 57, and the sensitivity and specificity were calculated to be 48% and 64%, respectively.

The typical onset time of PPS is often reported to be 2–3 weeks [8]. It usually occurs within the first month following cardiac surgery, and its occurrence after six months is extremely rare [16]. In our study, the time to drainage matching due to PPS after primary surgery was similar to that in existing studies.

Our study found an inverse correlation between body mass index (BMI) and the development of PPS, suggesting the potential protective effect of high BMI. This is in line with previous observations by Miller et al., who suggested that obesity-associated immunomodulatory effects, including elevated anti-inflammatory cytokines, such as IL-4 and IL-13, may reduce systemic inflammation [17]. However, studies have reported a significant association between high BMI and PPS [24]. Although our findings support the potential protective effect of BMI in the development of PPS, the precise mechanisms driving this association remain unclear and require further investigation of metabolic and immune pathways.

The type and extent of cardiac surgery are also important factors that affect the incidence of PPS. In a large epidemiological study by Lehto et al., aortic surgery was found to be one of the interventions with the highest risk of PPS [25]. Similarly, valve surgery has been reported to carry a higher risk of PPS than CABG [26]. The observed association between the type of surgery and a higher incidence of PPS seems to be mediated by pericardial damage rather than myocardial damage. This is because studies have not found any association between biomarkers of myocardial damage and PPS occurrence [27]. In our study, PPS development was significantly higher in patients who underwent aortic and valvular surgery. This finding is consistent with the pathophysiological mechanism in the literature that pericardial trauma is more common in aortic and valvular surgeries, and this triggers an autoimmune response and predisposes patients to PPS development [28]. On the other hand, although a lower risk has been reported in the literature for patients undergoing mitral valve replacement [17], no comparison was made between subgroups in our study because mitral and aortic valve operations were evaluated together. This may explain the partial discordance between our study and the previous findings. In addition, previous studies have emphasized that more complex surgeries often require longer cardiopulmonary bypass (CPB) times and that factors such as the prolonged exposure of the pericardial cavity to air activate immune mechanisms and predispose patients to PPS [29]. Although the CPB and ACC durations were similar between the groups in our study, it may be considered that this duration was longer, especially in patients who underwent aortic surgery, which may have contributed to the observed association.

In a sub-study of the COPPS study evaluating risk factors for PPS, pleural incision was also found to be an independent predisposing factor for this syndrome [30]. However, in our cohort, chest tube placement, used as a surrogate for pleural manipulation, showed no significant difference between the groups. This suggests that pleural incision alone may be insufficient to provoke PPS in the absence of other contributors, such as pericardial trauma or complex surgery.

Current guidelines recommend the use of nonsteroidal anti-inflammatory drugs (NSAIDs) in combination with colchicine for the treatment of PPS [14]. However, in severe cases, the invasive drainage of pericardial or pleural effusion may be required. In the literature, such invasive interventions have been associated with increased morbidity [16], prolonged hospitalization, and repeated hospitalizations [24,30]. As expected, the length of hospital stay and readmission rates were significantly higher in the PPS group. Mortality risk was higher in the PPS group. However, this difference was not statistically significant. These findings clearly demonstrated the clinical burden and morbidity associated with PPS. This finding is consistent with those reported in the literature [31,32]. In addition, the fact that INR levels and the frequency of anticoagulant use did not show a significant difference in the PPS group (Table 4) supports the idea that inflammatory processes are the dominant pathophysiological mechanism rather than bleeding tendency.

The autoimmune nature of PPS underscores the need for reliable biomarkers. Although CRP and ESR are commonly elevated, their diagnostic utility is limited by their poor specificity in the postoperative setting [24]. In our study, we aimed to fill this gap using more complex biomarkers (SII, SIRI, NLR, and MLR). These indices may offer superior specificity for identifying high-risk patients because they integrate the multiple cellular components of the immune response. Specific studies examining the SII and SIRI values in PPS are limited. In general, these indices are elevated when inflammatory processes are active. As PPS is characterized by the inflammation of the pericardium and a systemic inflammatory response, increased SII and SIRI values are expected in these patients. Both the SII and SIRI are based on neutrophil and lymphocyte ratios and are associated with inflammation. The SII is related to the balance between inflammatory cells (neutrophils and platelets) and immune cells (lymphocytes). SIRI provides a broader perspective on inflammatory processes, considering that there are more monocytes [33]. Both indices can be used to determine the inflammatory burden. However, the SIRI may be significantly increased in autoimmune and chronic inflammatory diseases owing to the role of monocytes in chronic inflammatory processes. The SII is a biomarker that reflects both thrombotic and systemic inflammation. In this study, both the SII and SIRI were significantly elevated in patients with PPS requiring invasive intervention. In particular, the SII showed a significant increase only at T4, suggesting that systemic inflammation increases with disease progression, better reflecting the inflammatory process that occurs in the advanced phase of the disease and which requires intervention. However, the fact that this increase was detected only in the T4 time interval limits the use of the SII as a predictive marker in earlier stages and limits its prognostic power. This may be related to the delayed role of platelets in immune modulation. Therefore, although the SII may be a robust marker of advanced PPS, it is less suitable for early prediction. However, the increase in the SIRI value at T3 and the persistence of this increase at T4 suggest that this marker may indicate early-stage inflammation and is advantageous for early diagnosis. ROC analysis showed that an SIRI cut-off of 3.34 yielded 81.3% specificity, supporting its use in early risk assessment. These findings suggest that the SII may be a more sensitive biomarker and that the SIRI may be a more specific biomarker. As a matter of fact, a similar result was reported in a prospective study of 440,000 participants focusing on the relationship between cancer cases and systemic inflammation [34].

Our results indicate that MLR, CRP, and SIRI levels are associated with the progression of PPS following surgical treatment, with the most significant differences observed on the 7th day post-surgery. MLR was the most sensitive marker in our analysis. Elevated MLR is a marker of inflammatory activity, accompanied by an increase in monocyte count and a decrease in lymphocyte count. Monocytes play a role in the late inflammatory response and tissue repair processes. MLR has been reported to be prominent in processes such as pericardial inflammation, cardiac fibrosis, and autoimmunity [35]. In addition, many studies have shown that MLR better reflects inflammation with autoimmune characteristics, and the sensitivity of these markers is high in late pericarditis such as PPS [1,35]. Our findings showed that high MLR levels, especially on postoperative day 7, were significantly associated with the development of PPS requiring surgical intervention. ROC analysis revealed 84% sensitivity with a cut-off value of 0.575, supporting its role as a practical screening tool in early postoperative follow-up. This index not only peaked before the second surgical procedure but also continued to increase in a statistically significant manner compared to control patients, indicating a sustained systemic inflammatory response. However, while MLR demonstrated high sensitivity in identifying patients at risk of PPS requiring surgical treatment, its moderate AUC (0.663) and limited specificity (45.3%) indicated that it should not be used alone in the clinical decision-making process. A multi-marker approach integrating MLR with SIRI and CRP levels may provide improved predictive accuracy and reduce false-positive rates.

In this study, the NLR did not change significantly at any time point, which is consistent with its known association with acute infection and trauma [36]. Since PPS is not an acute infection but the result of a delayed autoimmune process, it is consistent with the literature that NLR is not as discriminative as the other markers. Furthermore, since neutrophil predominance is common in all patients in the first few days after surgery, the specificity of the NLR may be reduced. In our study, CRP and WBC levels, two non-specific inflammatory markers, were significantly higher in the PPS group at T4. These markers are useful for confirming late-stage inflammation but lack specificity [37]. However, this elevation in the late stage (T4) may reflect persistent inflammation or pericardial effusion associated with immune response. Nevertheless, it should be noted that CRP and WBC counts alone have limited prognostic value in clinical decision-making. Their utility may increase when used in conjunction with other indices. Based on our findings, a multi-marker approach consisting of high CRP, MLR, and SIRI levels may improve prognostic accuracy and support clinical decision-making.

## 5. Study Limitations

This study has several limitations that should be considered when interpreting the results. First, its retrospective design inherently restricts the ability to establish causal relationships and increases susceptibility to selection bias. Second, the study was conducted in a single tertiary center, and although institutional guidelines ensured procedural consistency, variations in surgical techniques across different surgical teams may have influenced the postoperative outcomes. Similarly, echocardiographic assessments were performed by multiple clinicians, introducing the possibility of interobserver variability in imaging interpretation.

Another key limitation was the clinical heterogeneity of the study population. The inclusion of patients with different underlying cardiac pathologies, such as ischemic heart disease and structural valve disorders, may have contributed to the variability in the systemic inflammatory responses. Additionally, no formal statistical matching techniques (e.g., propensity score matching) were employed to balance baseline characteristics. Instead, blinded randomization and stratification based on the surgery type were used to mitigate confounding factors.

Another limitation relates to the inclusion of patients who had already received medical treatment with colchicine and/or indomethacin at the time of a suspected PPS. Although these patients ultimately required surgical intervention owing to disease progression, the prior use of anti-inflammatory therapy may have modulated biomarker levels, potentially confounding the interpretation of inflammatory trends.

While inflammatory biomarkers were collected at standardized intervals, the T4 time point posed a methodological challenge. In the PPS group, T4 represented the day before reintervention, varying between postoperative weeks 2 and 11, whereas in the control group, it corresponded to routine outpatient visits at predefined intervals. Although efforts were made to match the timing through institutional follow-up schedules, an exact temporal alignment could not be achieved. This temporal mismatch may have introduced bias in interpreting late-phase biomarker differences and should be considered when evaluating T4-related findings.

Additionally, we excluded patients who developed symptoms within the first 14 postoperative days under the assumption that early manifestations were likely related to acute postoperative inflammation rather than true PPS. However, we acknowledge that early-onset PPS may occur occasionally, as reported in the literature.

Finally, the generalizability of our findings may be limited by the single-center nature of the study and the absence of external validation. Multicenter prospective studies with larger sample sizes and matched controls are warranted to confirm the predictive utility of systemic inflammatory indices in PPS.

## 6. Conclusions

This study demonstrates that systemic inflammatory indices, particularly SIRI, MLR, and CRP, hold significant promise as predictive biomarkers for the development and severity of PPS cases that require surgical intervention. Among these, MLR emerged as the most sensitive marker, whereas the SIRI showed strong early diagnostic specificity. These findings underscore the clinical utility of integrating such indices into routine postoperative monitoring, particularly in the first week following surgery when classical inflammatory markers lose their discriminatory power. The observed dynamic elevation patterns, especially the early rise in the SIRI and MLR, may allow for the timely identification of high-risk patients and early initiation of anti-inflammatory therapies, potentially reducing the need for invasive intervention. Incorporating the SIRI, MLR, and CRP into post-cardiac surgery surveillance protocols may represent a meaningful advancement in the risk stratification and personalized management of PPS.

## Figures and Tables

**Table 1:** Preoperative patient characteristics.

		Control Group				PPS Group				*p*
		Mean ± Sd			Median	Mean ± Sd			Median	
Age		60.00	±	10.89	61.00	54.88	±	14.33	59.00	***0.025***
**Sex**										
Male	*n*/%	55		73.33%		51	68.00%			0.473
Female	*n*/%	20		26.67%		24	32.00%			
Weight (kg)		80.88	±	11.92	79.00	75.41	±	11.54	76.00	***0.010***
BMI		28.26	±	4.39	27.94	26.43	±	4.57	25.34	***0.004***
EF (preoperative)		54.95	±	8.77	58.00	55.79	±	7.91	60.00	0.666
HT		22		29.33%		19	25.33%			0.444
DM		13		17.33%		16	21.33%			0.535
HL		16		21.33%		20	26.67%			0.444
PAD		8		10.67%		10	13.33%			0.615
CRI		4		5.33%		6	8.00%			0.513
COPD		8		10.67%		4	5.33%			0.229
Smoking		12		16.00%		9	12.00%			0.48
Redo Surgery *		4		5.33%		5	6.67%			0.731
**ABO group**										0.977 *
A Rh(+)		28		37.33%		32	42.67%			
A Rh(-)		4		5.33%		2	2.67%			
B Rh(+)		7		9.33%		7	9.33%			
B Rh(-)		3		4.00%		3	4.00%			
AB Rh(+)		4		5.33%		2	2.67%			
AB Rh(-)		1		1.33%		1	1.33%			
0 Rh(+)		24		32.00%		23	30.67%			
0 Rh(-)		4		5.33%		5	6.67%			

BMI, body mass index; HT, hypertension; EF, ejection fraction; DM, diabetes mellitus; HL, hyperlipidemia; PAD, peripheral arterial disease; COPD, chronic obstructive pulmonary disease; CRI, Chronic Renal Injury. Statistical significance was set at the 95% confidence level (*p* = 0.05).* Redo surgery refers to patients who underwent a second cardiac operation due to causes unrelated to PPS, such as prosthetic valve dysfunction or graft failure.

**Table 2:** Intraoperative variables and surgical characteristics of the study population.

		Control Group					PPS Group					*p*	
		Mean		±	Sd	Median	Mean		±	Sd	Median		
**Intraoperative data**													
	Total CPB time (min)	117.18		±	22.72	115.00	121.89		±	25.27	125.00	0.141	
	ACC time (min)	84.97		±	17.15	85.00	80.72		±	20.51	80.00	0.130	
			***n***		**%**			***n***		**%**			
**Surgical procedure**													
	CABG		7		9.33%			6		8.00%		***0.045***	
	AVR		14		18.67%			10		13.33%			
	MVR		11		14.67%			10		13.33%			
	AVR+MVR+TVR (or ring annuloplasty)		6		8.00%			3		4.00%			
	AVR+MVR		6		8.00%			9		12.00%			
	MVR+TVR (or ring annuloplasty)		7		9.33%			3		4.00%			
	MVR+CABG		8		10.67%			3		4.00%			
	Aortic surgery		11		14.67%			29		38.67%			
	AVR+CABG		5		6.67%			2		2.67%			
**Cardiac surgical procedure**												0.620	
	Thoracotomy		1		1.33%			3		4.00%			
	Sternotomy		74		98.67%			72		96.00%			
**Chest tube localization**												0.518	
	Mediastinum		35		46.67%			30		40.00%			
	Mediastinum + left thorax		20		26.67%			18		24.00%			
	Mediastinum + right thorax		14		18.67%			16		21.33%			
	Mediastinum + left thorax + right thorax		6		8.00%			11		14.67%			

PPS, postpericardiotomy syndrome; ACC, aortic cross-clamp; CPB, cardiopulmonary bypass. CABG, coronary artery bypass graft; AVR, aortic valve replacement; MVR, mitral valve replacement; TVR, tricuspide valve replacement.

**Table 3:** The early postoperative outcomes and in-hospital course of patients in the control and PPS groups.

		Control Group				PPS Group				*p*
		Mean ± Sd			Median	Mean ± Sd			Median	
**Postoperative variables**										
	Mean ICU stay (h)	37.60	±	23.45	24.00	44.32	±	32.95	24.00	0.397
	Intubation time (h)	11.73	±	15.46	7.00	14.12	±	17.89	8.00	0.108
	Mean hospital stay	8.68	±	2.74	8.00	10.32	±	3.14	10.00	***0.001***
	Drainage (mL)	626.00	±	254.07	550.00	686.00	±	246.81	650.00	0.062
	EF (postoperative)	50.72	±	7.52	50.00	50.39	±	7.91	50.00	0.667
**Blood products**										
	Transfused ES (unit)	1.99	±	1.13	2.00	2.16	±	0.96	2.00	0.201
	Transfused FFP (unit)	2.81	±	1.00	3.00	2.67	±	1.13	3.00	0.425
	Transfused TS (unit)	0.61	±	1.47	0.00	0.67	±	1.53	0.00	0.828
**Postoperative complications**		***n***		**%**		***n***		**%**		
	Renal injury	10		13.33%		8		10.67%		0.615
	Dialysis	2		2.67%		1		1.33%		0.500 *
	Liver injury	5		6.67%		7		9.33%		0.547
	Stroke	2		2.67%		4		5.33%		0.681 *
	MOPPS	2		2.67%		5		6.67%		0.442
	Pneumonia	4		5.33%		9		12.00%		0.147
	LCOS	4		5.33%		6		8.00%		0.513
	Re-hospitalization	4		5.33%		17		22.67%		***0.002***
	Mortality	1		1.33%		6		8.00%		0.058

EF, ejection fraction; ICU, intensive care unit; ES, erythrocyte suspension; FFP, fresh frozen plasma; TS, thrombocyte suspension; MOPPS, multiple organ dysfunction syndrome; LCOS, low cardiac output syndrome. * Fisher’s exact test was used instead of the chi-square test due to low expected cell counts.

**Table 4:** Comparison of anticoagulant and antiplatelet strategies and mean INR levels before PPS surgery.

			Control Group				PPS Group				*p*
			Mean ± Sd			Median	Mean ± Sd		Median		
Mean INR before PPS surgery			2.52	±	0.48	2.40	2.87	±	0.93	2.60	0.114
Postoperative anticoagulant/antiplatelet use											
	Warfarin	*n*/%	47		59.49%		41		47.67%		0.32
	ASA	*n*/%	11		13.92%		16		18.60%		0.288
	Klopidogrel	*n*/%	7		8.86%		3		3.49%		0.190
	DAPT	*n*/%	6		7.59%		12		13.95%		0.132
	TT	*n*/%	4		5.06%		9		10.47%		0.147
	DOAC	*n*/%	4		5.06%		5		5.81%		0.731

ASA, acetyl salicylic acid; DAPT, dual antiplatelet (ASA+Klopidogrel); TT, triple therapy; DOAC, direct oral anticoagulant.

**Table 5:** Comparison of surgical techniques for PPS: drainage methods and frequency of pericardiopleural window.

Drainage MethoPPS		*n*	%	*p*
	Median sternotomy	26	34.67%	***<0*** ***.001***
	Thoracotomy (right/left)	37	49.33%	
	Subxiphoid incision	5	6.67%	
	Pericardiocentesis (ECHO-guided)	7	9.33%	
**PPW in PPS**				
	PPW	33	44.00%	

PPW, pericardiopleural window.

**Table 6:** Temporal variation in inflammatory marker levels at four postoperative time points in patients with and without postpericardiotomy syndrome.

		Control Group							PPS Group							*p*
		Mean ± Sd			Median	Min	–	Max	Mean ± Sd			Median	Min	–	Max	
**SII** (Systemic Inflammation Index)	Preoperative (T1)	520.89	±	173.49	481.14	306.84	-	1254.40	608.89	±	270.03	562.67	230.88	-	1738.09	0.073
	Postoperative day 1 (T2)	1482.56	±	1051.41	1131.58	523.33	-	7605.53	1468.86	±	1207.35	1240.80	385.55	-	7093.41	0.759
	Postoperative day 7 (T3)	1017.93	±	655.65	887.03	61.71	-	3884.51	1187.23	±	947.14	931.30	178.07	-	4637.39	0.551
	Before PPS surgery (T4)	654.17	±	275.13	583.63	314.72	-	1550.71	771.17	±	364.30	672.10	272.37	-	2216.86	***0.020***
	*p* (in-group)	<0.001							<0.001							
**SIRI** (Systemic Inflammation Response Index)	Preoperative (T1)	1.07	±	0.35	1.01	0.59	-	2.91	1.19	±	0.66	1.00	0.49	-	3.90	0.762
	Postoperative day 1 (T2)	4.97	±	2.17	4.32	2.03	-	13.81	4.78	±	2.36	4.15	1.35	-	13.81	0.327
	Postoperative day 7 (T3)	2.78	±	2.09	2.40	0.93	-	14.80	3.12	±	1.63	2.68	0.87	-	8.32	***0.036***
	Before PPS surgery (T4)	1.82	±	0.78	1.57	0.97	-	4.57	2.00	±	0.89	1.76	1.06	-	6.67	***0.064***
	*p* (in-group)	<0.001							<0.001							
**NLR** (Neutrophil to Lymphocyte Ratio	Preoperative (T1)	2.32	±	0.65	2.22	1.28	-	5.60	2.67	±	1.14	2.26	1.41	-	7.79	0.194
	Postoperative day 1 (T2)	7.67	±	2.74	7.05	4.29	-	18.50	7.48	±	2.76	6.93	3.47	-	18.50	0.693
	Postoperative day 7 (T3)	5.26	±	2.87	4.57	1.90	-	20.55	5.04	±	2.20	4.78	1.90	-	12.24	0.845
	Before PPS surgery (T4)	2.68	±	1.02	2.38	1.11	-	6.01	2.87	±	1.22	2.63	1.28	-	9.01	0.195
	*p* (in-group)	<0.001							<0.001							
**PLR** (Platelet-to-lymphocyte ratio)	Preoperative (T1)	139.87	±	40.52	129.71	83.11	-	320.00	144.31	±	43.29	139.11	45.27	-	265.22	0.397
	Postoperative day 1 (T2)	140.18	±	70.37	121.80	50.56	-	461.17	135.02	±	61.06	122.13	53.62	-	372.36	0.888
	Postoperative day 7 (T3)	138.38	±	74.82	123.20	10.71	-	400.76	159.65	±	94.27	135.22	40.95	-	428.43	0.322
	Before PPS surgery (T4)	165.97	±	49.57	162.94	86.10	-	308.79	191.25	±	60.86	177.21	61.62	-	490.74	***0.002***
	*p* (in-group)	<0.001					-		<0.001					-		
**MLR** (Monocyte-to-lymphocyte ratio	Preoperative (T1)	0.29	±	0.09	0.26	0.15	-	0.58	0.27	±	0.09	0.25	0.16	-	0.56	0.231
	Postoperative day 1 (T2)	0.48	±	0.12	0.44	0.27	-	0.78	0.46	±	0.13	0.44	0.18	-	0.78	0.391
	Postoperative day 7 (T3)	0.36	±	0.12	0.33	0.19	-	0.85	0.43	±	0.14	0.40	0.18	-	0.85	***0.001***
	Before PPS surgery (T4)	0.46	±	0.12	0.44	0.26	-	0.73	0.51	±	0.16	0.47	0.29	-	1.15	0.106
	*p* (in-group)	<0.001							<0.001							
**CRP** (C-reactive protein)	Preoperative (T1)	5.82	±	2.93	5.90	1.00	-	11.10	6.35	±	2.79	6.30	0.90	-	11.40	0.278
	Postoperative day 1 (T2)	93.75	±	76.83	57.26	23.15	-	321.00	100.69	±	79.97	57.29	23.15	-	381.00	0.381
	Postoperative day 7 (T3)	20.50	±	19.83	15.00	1.00	-	122.00	30.82	±	31.07	18.31	1.25	-	123.00	0.093
	Before PPS surgery (T4)	17.74	±	15.46	12.50	4.00	-	78.70	32.49	±	21.72	31.90	3.00	-	94.00	***0.000***
	*p* (in-group)	<0.001							<0.001							
**WBC** (White blood cell)	Preoperative	7.98	±	3.43	6.96	3.70	-	21.95	7.29	±	3.32	6.43	3.45	-	21.70	0.087
	Postoperative 1th day	14.39	±	4.15	13.72	5.11	-	29.44	15.11	±	5.32	14.70	5.97	-	35.97	0.501
	Postoperative 7th day	11.28	±	4.52	9.94	4.80	-	26.67	12.24	±	4.02	12.22	4.98	-	25.65	***0.040***
	Before PPS surgery	7.63	±	3.66	7.11	2.33	-	25.85	9.97	±	4.07	9.25	5.08	-	29.64	***0.000***
	*p* (in-group)	<0.001							<0.001							

SII, Systemic Inflammation Index; SIRI, Systemic Inflammation Response Index; PLR, Platelet-to-lymphocyte ratio; MLR, Monocyte-to-lymphocyte ratio; CRP, C-reactive protein; WBC, white blood cell.

**Table 7:** Changes in routine biochemical markers across time points in patients with and without postpericardiotomy syndrome.

		Control Group				PPS Group				*p*
		Mean ± Sd			Median	Mean ± Sd			Median	
Preoperative (T1)	Urea (mg/dL)	40.87	±	23.57	35.00	42.29	±	26.04	35.00	0.914
	Creatinine (mg/dL)	1.10	±	0.99	0.88	1.08	±	0.81	0.88	0.571
	LDH (U/Lt)	234.64	±	87.84	228.00	235.07	±	85.97	216.00	0.697
	AST (U/Lt)	29.45	±	24.41	22.00	27.07	±	21.80	22.00	0.589
	ALT (U/Lt)	34.23	±	49.04	20.00	28.68	±	38.81	20.00	0.763
	Albumin	38.07	±	8.59	42.00	37.11	±	8.61	40.00	0.457
Postoperative day 1 (T2)	Urea (mg/dL)	58.48	±	30.38	49.00	55.08	±	28.11	49.00	0.453
	Creatinine (mg/dL)	1.14	±	0.71	0.90	1.07	±	0.65	0.88	0.517
	LDH (U/Lt)	453.25	±	159.81	434.00	450.93	±	160.21	438.00	0.998
	AST (U/Lt)	104.21	±	103.27	70.00	100.33	±	97.31	73.00	0.886
	ALT (U/lt)	51.55	±	75.01	32.00	58.31	±	85.31	32.00	0.645
	Albumin	31.19	±	4.56	31.00	31.45	±	4.35	31.00	0.710
Postoperative day 7 (T3)	Urea (mg/dL)	51.28	±	36.82	41.00	51.77	±	40.29	41.00	0.979
	Creatinine (mg/dL)	1.23	±	0.88	0.98	1.25	±	0.89	0.98	0.722
	LDH (U/Lt)	376.44	±	181.57	357.00	399.87	±	166.63	384.00	0.333
	AST (U/Lt)	63.64	±	131.05	35.00	64.67	±	178.60	38.00	0.665
	ALT (U/Lt)	50.67	±	57.07	38.00	53.52	±	56.66	40.00	0.462
	Albumin	33.08	±	4.53	32.00	32.49	±	4.34	32.00	0.455
Pre-intervention for PPS (T4)	Urea (mg/dL)	55.08	±	47.32	44.00	53.97	±	47.52	41.00	0.769
	Creatinine (mg/dL)	1.31	±	1.25	0.89	1.25	±	1.16	0.89	0.816
	LDH (U/Lt)	283.09	±	147.65	256.00	317.71	±	132.29	313.00	***0.020***
	AST (U/Lt)	44.72	±	77.36	30.00	45.63	±	77.50	30.00	0.961
	ALT (U/Lt)	47.03	±	45.41	36.00	46.17	±	44.48	35.00	0.955
	Albumin	34.24	±	5.44	36.00	34.27	±	5.22	36.00	0.904

LDH, lactate dehydrogenase; AST, aspartate aminotransferase; ALT, alanine aminotransferase.

**Table 8:** Univariate and multivariate logistic regression analyses of postoperative biomarkers for predicting the development of postpericardiotomy syndrome.

	Univariate Model				Multivariate Model *			
	OR	95% C. I		*p*	OR	95% C. I		*p*
Age	0.968	0.940	0.994	***0.017***	0.967	0.940	0.995	***0.012***
BMI	0.911	0.845	0.983	***0.016***				
**Postoperative day 7**								
Monocyte	290.820	18.500	4570.940	***<0.001***	340.315	18.995	6100.153	***0.00***
SII	1.000	1.000	1.001	0.209				
SIRI	1.106	0.921	1.329	0.280				
Monocyte–lymphocyte (MLR)	59.036	3.985	874.601	***0.003***				
CRP	1.018	1.003	1.032	***0.015***	1.016	1.001	1.031	***0.04***

SII, Systemic Inflammation Index; SIRI, Systemic Inflammation Response Index; MLR, Monocyte-to-lymphocyte ratio; CRP, C-reactive protein. * In the multivariate analysis, variables were reduced using the Forward Likelihood Ratio (LR) method.

**Table 9:** Diagnostic performance of selected biomarkers for predicting surgically significant postpericardiotomy syndrome based on ROC curve analysis (postoperative day 7).

Variables	AUC	*p*	95% CI		Cut-Off	Sensitivity	Specificity	Positive Predictive Value	Negative Predictive Value
Age	0.591	0.055	0.5	0.68	57	48.00%	64.00%	57.14%	5.17%
SIRI	0.599	***0.036***	0.51	0.69	3.34	32.00%	81.33%	63.16%	54.46%
CRP	0.604	***0.028***	0.513	0.695	16.470	60.00%	61.33%	60.81%	60.53%
MLR	0.663	***0.001***	0.575	0.750	0.310	84.00%	45.33%	60.58%	73.91%

AUC, area under the curve; CI: confidence interval; SIRI: Systemic Inflammatory Response Index; MLR: Monocyte-to-Lymphocyte Ratio; CRP: C-reactive protein.

## Data Availability

Raw data supporting the conclusions of this study will be made available by the authors upon request.
